# The Influence of Temperature and Host Gender on Bacterial Communities in the Asian Citrus Psyllid

**DOI:** 10.3390/insects12121054

**Published:** 2021-11-25

**Authors:** Rui-Xu Jiang, Feng Shang, Hong-Bo Jiang, Wei Dou, Tomislav Cernava, Jin-Jun Wang

**Affiliations:** 1 Key Laboratory of Entomology and Pest Control Engineering, College of Plant Protection, Academy of Agricultural Sciences, Southwest University, Chongqing 400715, China; b1995031@email.swu.edu.cn (R.-X.J.); fengshang94@swu.edu.cn (F.S.); jhb8342@swu.edu.cn (H.-B.J.); douwei80@swu.edu.cn (W.D.); 2 Institute of Environmental Biotechnology, Graz University of Technology, 8010 Graz, Austria; tomislav.cernava@tugraz.at

**Keywords:** symbionts, temperature, *Diaphorina citri*, 16S sequencing

## Abstract

**Simple Summary:**

*Diaphorina citri* is an important natural vector for the Huanglongbing pathogen, which causes destructive damage to citrus production. The temperature and gender are critical abiotic and biotic factors affecting insect physiology as well as the symbiont abundance. Nevertheless, how temperature and gender affect the bacterial communities present in *D. citri* is still unclear. This study used high-throughput sequencing of 16S ribosomal RNA amplicons to identify amplicon sequence variants in *D. citri*. The dominant phylum was Proteobacteria, and *Candidatus Profftella* and *Wolbachia* were the dominant taxa in all groups. Furthermore, under a high-temperature treatment, *Profftella* was the prevalent symbiont in females, but *Wolbachia* had a higher abundance in males. In males, *Profftella* was more abundant under low-temperature treatments than high-temperature treatments. In contrast, *Wolbachia* showed a higher abundance under high-temperature treatments than under low-temperature treatments. The results will provide a new vision for understanding the co-adaptation of *D. citri* and its symbionts to environmental stresses.

**Abstract:**

The Asian citrus psyllid, *D. citri* Kuwayama is the primary vector for *Candidatus Liberibacter asiaticus* (CLas), which causes a destructive disease in citrus plants. Bacterial symbionts are important determinants of insect physiology, and they can be impacted by many external factors. Temperature is an important abiotic factor affecting insect physiology, and it is also known that differences in symbiont proportions may vary in different insect genders. To date, it is unclear how the symbionts of *D. citri* are affected by temperature and gender. This study used high-throughput sequencing of 16S ribosomal RNA amplicons to determine how temperature and gender affect the bacterial communities present in *D. citri*. We identified 27 amplicon sequence variants (ASVs) belonging to 10 orders, seven classes, and five phyla. The dominant phylum was Proteobacteria (99.93%). Other phyla, including Firmicutes, Bacteroidota, Deinococcota, Cyanobacteria, and Actinobacteriota, were less abundant (<0.1%). *Profftella* (71.77–81.59%) and *Wolbachia* (18.39–28.22%) were the predominant taxa in all samples. Under high-temperature treatment, *Profftella* was more common in females, while *Wolbachia* had a higher abundance in males. In males, *Profftella* was more abundant under low-temperature treatments than under high-temperature treatments. In contrast, *Wolbachia* showed a higher abundance under high-temperature treatments than under low-temperature treatments. An RT-qPCR (quantitative real-time PCR) approach confirmed the results obtained with high-throughput DNA sequencing. Our results provide a basis for understanding the co-adaptation of *D. citri* and its symbionts to environmental temperature stress.

## 1. Introduction

Various bacterial symbionts have colonized and become essential components of their host’s physiology in insects [1,2]. For example, symbionts synthesize nutritional proteins and essential amino acids and provide them to their hosts. These substances often cannot be synthesized by a host or are present only in insufficient levels in the environment. *Buchnera aphidicola* is a common primary symbiont of aphids, and it can secrete essential amino acids required by the host to maintain its normal physiological activity [3]. Symbionts can also help their host adapt to different stress factors [4,5]. *Hylobius abietis* harbors specific gut microbiota that can degrade conifer diterpenes to avoid the exposure of the host to toxic compounds, thus improving the host’s fitness [6]. Members of the genus *Wolbachia* are facultative bacteria, infecting arthropods and filarial nematodes [7]. They can regulate their host’s gene expression programs, in addition to altering other functions, to increase the host’s stress tolerance [8].

*D. citri* Kuwayama (Hemiptera: Liviidae) is an important citrus pest that occurs globally, including in all of the major citrus production areas in Asia and the Americas [9,10,11,12]. The psyllid can transmit the phloem-limited bacterium CLas, which causes Huanglongbing disease (HLB; also known as ‘citrus tree cancer’) [13]. HLB can substantially reduce citrus production and cause tree death. Although novel peptide based approaches to minimizing HLB transmission are in the pipeline [14], the current management of this disease involves the complete removal of diseased trees from orchards.

Global climate change is an important factor that has often been linked to such diseases’ occurrence, especially in the temperature, which has been regarded as the dominant abiotic factor affecting insects. The temperature can directly affect insect development, survival, range, and abundance [15]. Some insects have developed specific strategies to adapt to temperature changes [16]. Additionally, symbionts can support their host to withstand temperature stress. For example, the maternally inherited bacterium *Cardinium* increases the longevity of *Bemisia tabaci* and adjusts oviposition periods to resist unfavorable conditions [17]. With environmental temperatures increasing globally due to climate change, *D. citri* habitats have also expanded. Based on previous studies, the best season to curb the *D. citri* population is winter [18]. However, this psyllid control strategy may become ineffective, because the mean temperatures during winter are increasing due to global warming. Determining symbiont dynamics in *D. citri* under different temperatures can improve our understanding of their interplay and facilitate the development of improved pest management strategies.

High-throughput DNA sequencing technology provides a method to identify and characterize complex bacterial communities [19]. The sequencing of 16S ribosomal RNA gene fragments has been implemented to describe the bacterial communities present in a distinct sample [20]. This method has already been used in many studies to analyze insect symbionts [21,22]. Thus, we examined psyllid bacterial community dynamics under different temperatures and host genders and used RT-qPCR to validate the results of high-throughput sequencing.

## 2. Materials and Methods

### 2.1. Psyllid Samples

*D. citri* was initially collected from HLB-free citrus groves in 2012 in Jiangxi province, China. It was continuously reared on three-year-old *Murraya paniculata* under controlled laboratory conditions (26 ± 3 °C; 50 ± 10% relative humidity; Light: Dark = 14:10) for more than 45 generations.

### 2.2. Temperature Treatments

To establish a reasonable temperature gradient, we assessed local annual average temperature data (www.cma.gov.cn, (accessed on 28 June 2021)) and published articles [23]. Based on these data, three representative temperatures were selected as treatment conditions (15 °C, 26 °C, and 42 °C). Treatment groups of psyllids were released on mature leaves of *Murraya paniculata* in net cages, then incubated in controlled incubators (Ningbojiangnan instrument company, Ningbo, China) set at the different temperatures. 

### 2.3. Psyllid Collection

Five-day-old adult virgin male and female psyllids were used for sample preparation. Every treatment was conducted for 6 h at different temperatures, and four biological replicates were used, with each replicate including 20 male or female *D. citri* adults that were collected using sterile centrifuge tubes. All of the samples were stored in 75% ethyl-alcohol at –20 °C until DNA extraction.

### 2.4. DNA Extraction

All of the psyllid samples were washed three times with sterile water before the DNA extraction. The DNA extraction was performed according to the manufacturer’s instructions for the QIAGEN DNeasy Kit (QIAGEN, Hilden, Germany). All of the DNA samples were quality-checked, and A260/A280 and A260/A280 values of 1.95 to 2.10 were used to qualify samples for further processing. DNA concentrations were quantified with a NanoDrop 2000 spectrophotometer (Thermo Fisher Scientific, Wilmington, DE, USA). All of the DNA extracts were stored at −20 °C before a polymerase chain reaction (PCR) was performed.

### 2.5. PCR Amplification and Sequencing of 16S rRNA Amplicons

To study temperature effects on the microbial community composition of *D. citri*, 16S rRNA amplicon sequencing was performed. Bacterial 16S rRNA gene fragments (V3–V4) were amplified from extracted DNA samples using primers 338F (5′-ACTCCTACGGGAGGCAGCAG-3′) and 806R (5′-GGACTACHVGGGTWTCTAAT-3′), and the PCR was carried out in 20-μL reaction mixes containing 4 μL of 5 × FastPfu buffer, 2 μL of 2.5 mM dNTPs, 0.8 μL of each primer (5 μmol), 0.2 μL of BSA, 10 ng of DNA. The PCR cycling parameters were 95 °C for 3 min, 27 amplification cycles at 95 °C for 30 s, 55 °C for 30 s, and 72 °C for 45 s, with a 10-min final extension at 72 °C. Sterile water was used as a negative control for all PCRs. Subsequently, PCR products were visualized on 2% agarose gels. High throughput sequencing was conducted using the Illumina Miseq platform (Illumina, California, CA, USA) and the read length used a PE300 manufactured by Shanghai Majorbio Bio-Pharm Technology Co. Ltd. (Shanghai, China).

### 2.6. Bioinformatic Processing of Amplicon Datasets

Paired-end reads were merged using FLASH (v1.2.7) [24] based on unique barcodes. Subsequently, reads were truncated by removing barcode and adapter sequences and quality-filtered using fastp (0.19.6) [25]. Amplicon sequence variants (ASVs) were identified using the QIIME2 (version 2020.2) [26] pipeline with recommended parameters. This process generated de-noised high-quality sequences generated by DADA2 with single-nucleotide resolution based on the error profiles within samples [27]. Taxonomic assignments were made using the Naive Bayes consensus taxonomy classifier implemented in QIIME2 and the SILVA 16S rRNA database (v138). All of the processing steps for our 16S rRNA gene fragment library were performed on the free online Majorbio Cloud Platform (cloud.majorbio.com, (accessed on 11 December 2020)).

### 2.7. Statistical Analyses

The significance of differences in the alpha diversity was tested with the Kruskal-Wallis test, and the beta diversity was assessed by the similarities test (ANOSIM, analysis of similarities). Both were integrated in the QIIME 2 pipeline. The statistical significance of differences in qPCR data was assessed with a one-tailed Student’s *t* test (* *p* < 0.05; ** *p* < 0.01; *** *p* < 0.001) using SPSS version 16.0 (IBM, Armonk, NY, USA).

### 2.8. Phylogenetic Analysis

To investigate the evolutionary relationships of the identified ASVs in *D. citri*, the obtained 16S sequences (Table S1) were aligned using ClustalW with default settings [28]. A phylogenetic tree based on this alignment was constructed in MEGA v5.05 using the neighbor-joining method, and bootstrap values were calculated based on 1000 replicates [29].

### 2.9. Determination of Bacterial Abundance by Quantitative Real-Time PCR (RT-qPCR)

To confirm our amplicon sequencing results, an RT-qPCR approach was implemented to determine the relative abundance of each phylum. The primers used are listed in Table S2. Male and female psyllid adults were collected as described above. DNA was extracted using the QIAGEN Dneasy Kit (QIAGEN, Hilden, Germany) according to the manufacturer’s specifications with 10 biological replicates, and each replicate included 10 male or female *D. citri* adults. RT-qPCR was performed using a LightCycler^®^ 96 PCR detection System (Roche, Basel, Switzerland) with mixtures containing 5 μL of NovoStart SYBR qPCR SuperMix (Novoprotein, Shanghai, China), 0.5 μL of each primer (0.2 mM), 0.5 μL of template DNA, and 2.5 μL of nuclease-free water. Amplification was performed using the following conditions: 10 min at 95 °C, 40 cycles for 10 s at 95 °C, 20 s at 60 °C, and a final cycle for 20 s at 72 °C. Two reference genes, *GADPH* and *actin*, were used to normalize the 16S rRNA gene copy numbers using qBASE [30].

## 3. Results

### 3.1. Assessment of the Amplicon Dataset Obtained from D. citri

**Figure 1:**
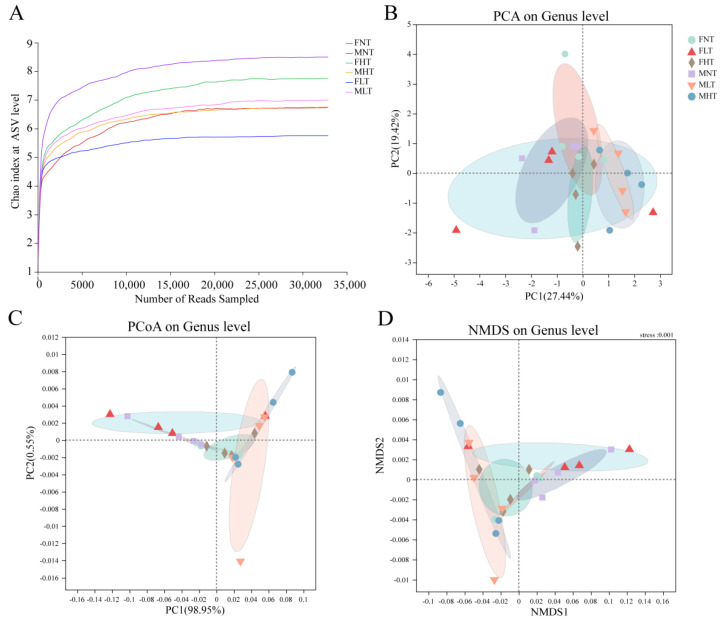
Rarefaction curves of α-diversity indices and β-diversity plots of bacterial communities in *D*. *citri* by temperature. (**A**) Chao I diversity index. Samples’ cluster analysis plot of the bacterial communities (**B**–**D**) of *D*. *citri* adults at different temperatures, at the genus level. (**B**) Principal components analysis (PCA). (**C**) Principal coordinates analysis (PCoA). (**D**) Non–metric multidimensional scaling (NMDS) analysis. The different color circles and triangles correspond to samples from *D. citri* adults. FNT and MNT: female and male under a normal temperature (26 °C); FHT and MHT: female and male under a high temperature (42 °C); FLT and MLT: female and male under a low temperature (15 °C). Every group used four biological replicates of *D. citri*.

The sequencing on the Illumina HiSeq 2500 platform (Illumina, California, CA, USA), targeting the V3-V4 hypervariable regions of the bacterial 16S rRNA gene in psyllids, yielded 5,824,440 raw reads (1,753,156,440 bp) in total. After quality-filtering, 601,640,661 bp of data were retained, with an average length of 423 bp (Table S3). Chao l rarefaction curves were used to estimate the within-sample diversity. The α-diversity indices plateaued at less than 32,000 reads sampled. This indicated that the sequencing performed was sufficient to assess the diversity within all samples (Figure 1A).

### 3.2. Taxonomic Composition of the Bacterial Community

The analysis of the bacterial community composition of *D. citri* indicated that Proteobacteria (99.93%) was the most dominant phylum. All other phyla (e.g., Firmicutes, Bacteroidota, Deinococcota, and Actinobacteriota) occurred in significantly lower abundances (<0.1%). Two classes of Proteobacteria accounted for a fraction of more than 0.1%. These classes were assigned to Gammaproteobacteria, with 72.68%, Oxalobacteraceae and Alphaproteobacteria, with 27.25%, and Anaplasmataceae (Table S4).

### 3.3. Bacterial Diversity of D. citri under Different Temperature Treatments

**Figure 2:**
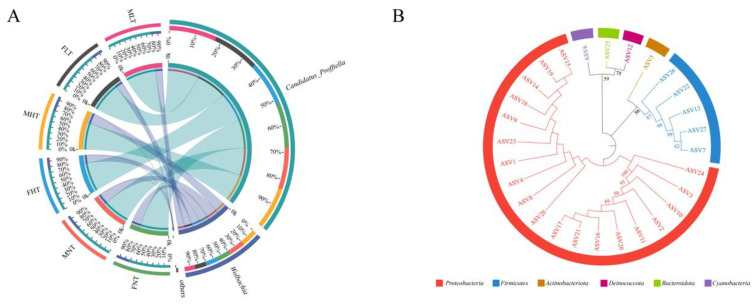
Impact of different temperature treatments of *D*. *citri* on the compositions of bacterial communities. (**A**) Chord diagram of relative abundance classified at the genus taxonomic level. (**B**) Phylogenetic analysis of the bacterial community identified in *D. citri* based on amplicon sequence variant (ASV) sequences. A tree was constructed using the neighbor–joining method via MEGA 5.05. Bootstrap support values for 1000 samples were provided on the branches (only showing values above 50%). The ASV sequences used for constructing the phylogenetic tree are listed in Table S2. FNT and MNT: female and male under a normal–temperature treatment (26 °C); FHT and MHT: female and male under a high–temperature treatment (42 °C); FLT and MLT: female and male under a low-temperature treatment (15 °C). Every group had four biological replicates of *D. citri*.

Three clustering algorithms, including principal components analysis (PCA), principal coordinate analysis (PCoA), and non-metric multidimensional scaling (NMDS) based on Bray-Curtis dissimilarities, were used to estimate the differences in these bacterial communities at the genus level after temperature treatments with male and female *D. citri* adults. Four biological replicates from females (or males) formed one group with three temperature treatments and showed a divergence in the genus-level clustering between the different temperature treatment groups (Figure 1B–D), and the bacterial structure varied at the genus level (Figure 2A).

Based on the DADA2 algorithm, 27 ASVs were identified from the three different temperature treatments. These 27 ASVs were assigned to 10 orders, 10 families, 10 genera, and 11 species based on our phylogenetic tree (Figure 2B). Among these, the high-temperature treatment groups showed a relatively lower bacterial diversity and contained 16 ASVs that were assigned to five genera, five families, five orders, five classes, and four phyla. Low-temperature treatment groups showed a moderate bacterial diversity, containing 18 ASVs assigned to six genera, six families, six orders, five classes, and four phyla. The normal-temperature groups harbored a relatively higher bacterial diversity than the high-temperature group and contained 24 ASVs assigned to nine genera, nine families, nine orders, seven classes, and five phyla (Table S5).

*Profftella* occurred in the highest proportion among the bacterial community in all samples. This order accounted for an average of 80.88% under the low-temperature treatment, 75.00% under the high-temperature treatment, and 73.64% under the normal-temperature treatment.

**Figure 3:**
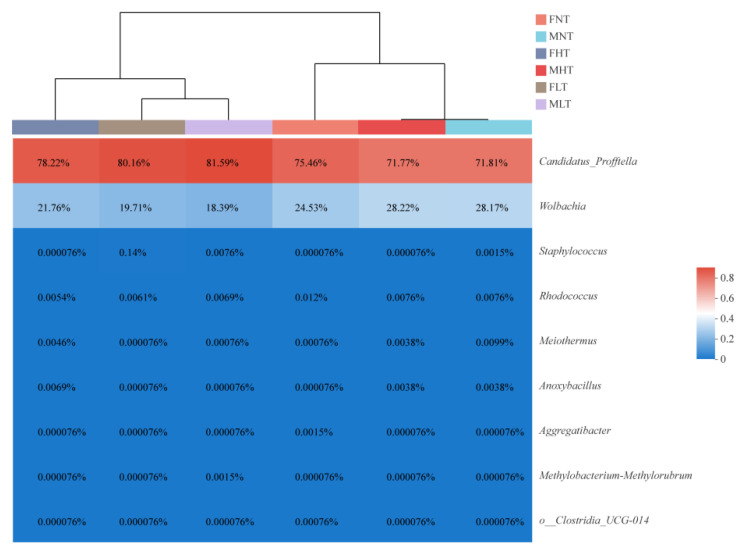
Impact of different temperature treatments of *D. citri* on the compositions of bacterial communities. The colors indicate the relative abundance, ranging from blue–gray (a lower relative abundance) to red–gray (a higher relative abundance). The dominant ASV proportion in male and female adults of *D. citri* under different temperature treatments and the relative abundances of the bacterial distributions of the top 50 abundant genera present in the microbial community are depicted. FNT and MNT: female and male under a normal–temperature treatment (26 °C); FHT and MHT: female and male under a high-temperature treatment (42 °C); FLT and MLT: female and male under a low–temperature treatment (15 °C). Every group had four biological replicates of *D. citri*.

The symbiont *Wolbachia* was detected at an average proportion of 19.05% under the low-temperature treatment, 24.99% under the high-temperature treatment, and 26.35% under the normal-temperature treatment (Figure 3). Other bacteria at the genus level were present in a lower abundance (less than 0.1%) in *D. citri* adults (Table S6).

### 3.4. Symbiotic Community Dynamics of D. citri Are Affected by Temperature and Gender

**Figure 4:**
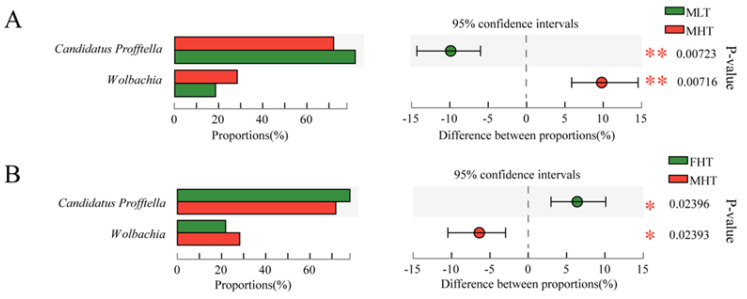
Comparison of the bacterial community in MLTs and MHTs and in FHTs and MHTs. (**A**) Males in low– and high–temperature treatments. (**B**) Females and males in high-temperature treatments. Student’s *t*-test was used to statistically determine the significant differences between males and females of *D. citri* under low–temperature or normal–temperature treatments (* *p* < 0.05; ** *p* < 0.01). FHT and MHT: female and male under high temperature (42 °C); MLT: male under low temperature (15 °C). Every group had four biological replicates of *D. citri*.

Sequencing data indicated that the relative abundance of the dominant bacteria, *Profftella* and *Wolbachia*, differed significantly under the tested temperatures (Figure 4A). At a low temperature, male adults showed a higher proportion of *Profftella* (mean = 81.59%) than at higher temperatures (mean = 71.77%). At a high temperature, male adults showed a higher proportion of *Wolbachia* (mean = 28.22%) than at low temperatures (mean = 18.39%) (Figure 3). Other treatment comparisons were not significantly different (Figures S1 and S2).

When genders were compared, the abundances of the dominant symbiotes, *Profftella* and *Wolbachia*, were significantly different (Figure 4B). At a high temperature, females (mean = 78.22%) showed a higher proportion of *Profftella* than males, and males (mean = 28.22%) showed a higher proportion of *Wolbachia* than females (Figure 3). Other treatment comparisons were not significantly different. 

### 3.5. RT-qPCR Validation

**Figure 5:**
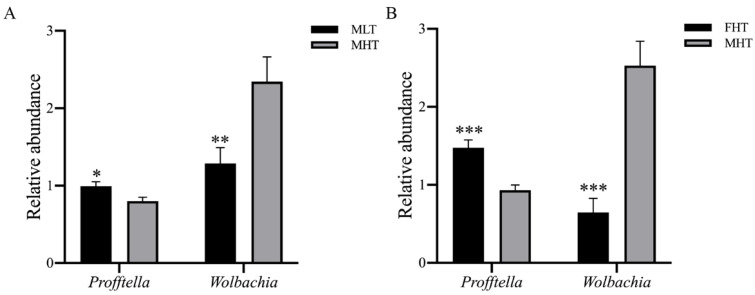
RT-qPCR validation of high-abundance bacteria (*Profftella* and *Wolbachia*) both in (**A**) MLT and MHT and (**B**) FHT and MHT, as well as a high relative abundance (>0.1%). The relative expression level of ASV 16S rRNA is an index of the bacterial abundance. Two reference genes, *GADPH* and *actin*, were used to normalize the 16S rRNA expression level in qBASE. The mean (±SE) expression level was based on 10 biological replicates. Significant differences between MLT and MHT and between FHT and MHT of *D. citri* were compared by Student’s *t*-test and are indicated by asterisks (* *p* < 0.05; ** *p* < 0.01; *** *p* < 0.001). FHT and MHT: female and male under a high-temperature treatment (42 °C); MLT: male under a low-temperature treatment (15 °C). Every group had four biological replicates of *D. citri*.

The two most abundant bacteria, *Profftella* and *Wolbachia*, for which amplicon sequencing indicated differential abundance in the different temperatures and gender groups assessed, were selected for further RT-qPCR confirmation. RT-qPCR results showed that *Profftella* was most abundant in the low-temperature-treated individuals. *Wolbachia* was most abundant in high-temperature-treated *D. citri* males (Figure 5A). Moreover, in these high-temperature treatments, *Profftella* had a relatively higher abundance in females, and *Wolbachia* had a higher abundance in males (Figure 5B). Overall, our RT-qPCR results were consistent with our amplicon sequencing data and two-factor analyses (Tables S7 and S8), confirmeing the temperature and gender-related dynamics of bacterial symbionts.

## 4. Discussion

In the present study, a targeted approach was implemented to assess whether and how the temperature stress affects bacterial symbionts of *D. citri*. Symbiotic microbes are known to be impacted by the environmental stress exposure of their hosts. Some of these have been previously shown to be involved in insect responses to the temperature, which can also affect the relationship between a host and its microbes [31]. Thermal changes can impact host’s metabolism and reduce the host’s survival and lifespan under extreme conditions [32]. To study the association between symbionts and temperature changes, the microbial community of a psyllid was evaluated at different temperatures by analyzing female and male hosts separately. We expected that when an insect host experiences temperature stress, it can lead to a fitness change of its symbionts. For example, heat stress led to the depletion of obligate endosymbionts (*Buchnera*), decreased fertility, and delayed developmental times in the pea aphid, *Acyrthosiphon pisum* [33]. Moreover, at high temperatures, the gut symbiont populations of stinkbugs were disturbed, leading to decreased host growth and reduced survival. The temperature can affect the stability of symbionts, and the subsequent symbiont instability can be the ‘Achilles’ heel’ of a host.

Symbiont and microbiome studies on *D. citri* are still relatively rare. Thus, detailed bacterial functions and the symbiont–host interplay in *D. citri* are still not fully resolved. Based on previous studies, *D. citri* has three endosymbionts from the egg stage until the adult stage, including the *Candidatus* species *Carsonella ruddii* and *Profftella* armature and *Wolbachia* [10]. The symbiont titer was shown to gradually increase with the development of *D. citri* until the adult stage. Female adult psyllids were shown to have increased symbiont titers before their egg laying [10,34]. In our study, the endosymbiont *Candidatus Carsonella ruddii* was not detected, while the two other symbionts were present. We speculated that this endosymbiont was not the dominant symbiont in *D. citri* in China.

*Profftella*, as a unique obligate symbiont in *D. citri* [35], can produce diaphorin, which is a polyketide toxic to various eukaryotic organisms, and can protect *D. citri* to avoid its predation [36]. Moreover, it can provide essential vitamins to *D. citri* to ensure its nutritional balance. Under the treatments conducted in the present study, *Profftella* occurred at higher proportions in *D. citri* females than males. Moreover, its proportion decreased in the high-temperature group but increased proportionally in the low-temperature group.

*Wolbachia* is a widespread facultative symbiont that is estimated to infect more than half of all insect species. It is involved in various important functions, such as male-killing, feminization, parthenogenesis, and cytoplasmic incompatibility [37], and has previously been exploited to curb dengue fever transmission between humans and mosquitoes [38]. *Wolbachia* can also be involved in other functions, such as supplying nutrition to a host to enhance the host’s reproduction [39]. In *D. citri*, a spanning analysis found that it can harbor four different strain types. Recent studies investigated the *Wolbachia* strain types in *D. citri* in global citrus colonies. Other studies reported that *Wolbachia* titers increased when healthy *D. citri* adults acquired CLas from a diseased plant [40,41].

In the present study, *Wolbachia* occurred in high proportions in all treatment groups. *Wolbachia* has been previously found to be highly temperature sensitive in many insect hosts [42], possibly affecting host–symbiont interactions. Moreover, it was observed that *D. citri* males had a higher heat resistance than females [43], but this biological phenomenon remains unexplained. Based on our results, we speculate that when the psyllid faces a high temperature stress, its symbiotic *Wolbachia* is activated. This might stimulate gene expression (e.g., heat shock proteins) [44] to assist the psyllid in maintaining a stable physiological function and improve the psyllid’s heat resistance. Our study only focused on *D. citri* symbiont dynamics under different temperatures and genders; therefore, a detailed examination remains to be done in future. A critical barrier facing mechanistic studies will be eliminating the specific symbionts in *D. citri* to obtain infected and uninfected psyllid strains. This will facilitate detailed assessments of how distinct symbionts might facilitate the survival and fitness of *D. citri* when exposed to environmental stress factors.

## 5. Conclusions

In summary, we assessed the microbial composition and population dynamics of *D. citri*. We studied its bacterial communities at different temperatures and differentiated between male and female hosts. The two known symbionts, *Profftella* and *Wolbachia*, were predominant in all of the samples. The proportions of these symbionts changed with the temperature, which provides evidence that this effect might be exploitable to reduce a psyllid’s fitness. Further work is required to elucidate the underlying mechanisms and functional implications of these observed symbiont dynamics.

## Data Availability

All data reported in this manuscript are available upon request.

## Supplementary Materials

The following are available online at https://www.mdpi.com/article/10.3390/insects12121054/s1: Table S1. The 16S sequences used for constructing the phylogenetic tree; Table S2. Primer sequences used for quantitative real-time PCR; Table S3. Overview of 16S rRNA gene fragment sequencing dataset obtained with *D*. *citri*; Table S4. Bacterial community composition (%) in *D*. *citri* at different taxonomic levels under different temperatures (only taxa with a relative abundance > 0.1% are shown); Table S5. Bacterial community composition in different samples of *D*. *citri*. Total counts are provided for different sample types at the specified taxonomic resolutions; Table S6. The bacterial community of *D*. *citri* at the different taxonomic level and their abundance in different temperature treatments; Table S7. The *Wolbachia* and *Profftella* proportion of male adults under the high and low-temperature treatments by Two-factor analyses; Table S8. The *Wolbachia* and *Profftella* proportion of male and females under the high-temperature treatments by Two-factor analyses; Figure S1. Comparison of the bacterial community dynamics with same-gender of *D*. *citri* in different temperature treatments; Figure S2. Comparison of the bacterial community dynamics of *D*. *citri* at the same temperature but different gender.
