# Supplementary material for: The Influence of Temperature and Host Gender on Bacterial Communities in the Asian Citrus Psyllid
Source: Insects. 2021 Nov 25;12(12):1054. doi: 10.3390/insects12121054 (PMC8704560; doi:10.3390/insects12121054)
Supplement: Supplementary file 1 [file insects-12-01054-s001.zip › insects-1449189-supplementary.pdf]

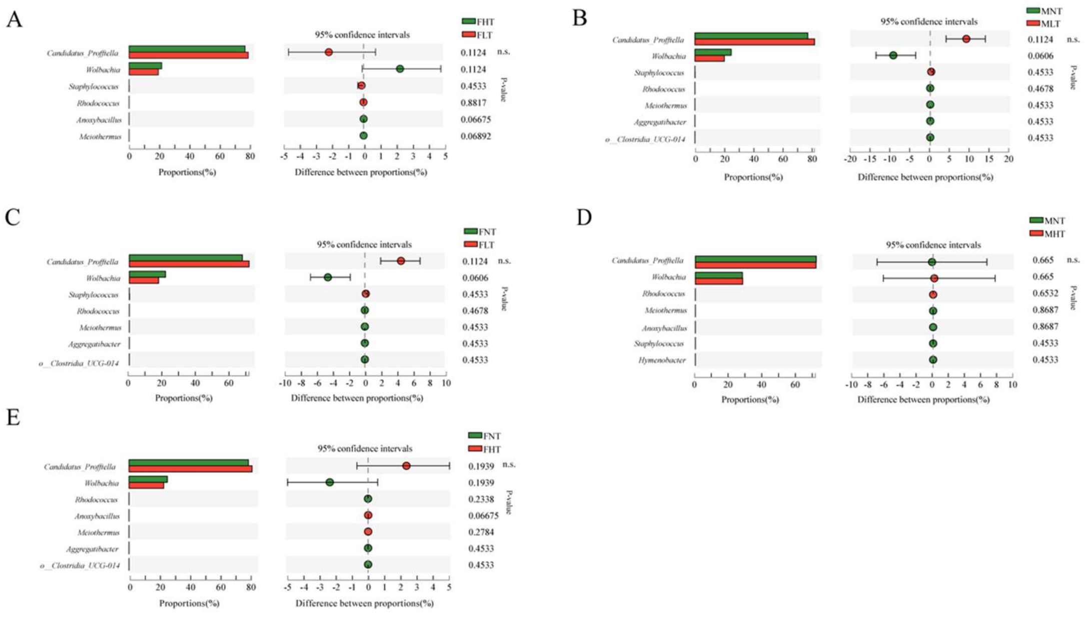

Figure S1. Comparison of the bacterial community dynamics with same-gender of *Diaphorina citri* in different temperature treatments. Student's t test was used to statistically determine the significant differences between males and females of *Diaphorina citri* in high–temperature, low–temperature, or normal–temperature (n.s. indicates no significance) treatments. FNT and MNT: female and male under normal temperature (26°C); FHT and MHT: female and male under high temperature (42°C); FLT and MLT: female and male under low temperature (15°C). Every group had four biological replicates of *D. citri*.

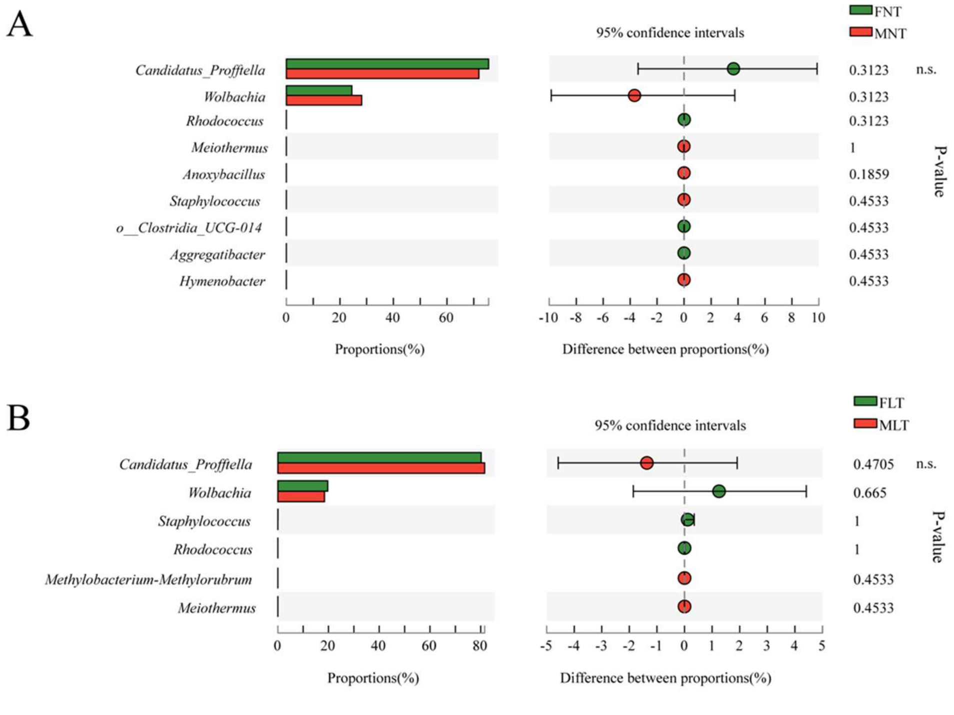

Figure S2. Comparison of the bacterial community dynamics of *Diaphorina citri* at the same temperature but different gender. Student's t test was used to statistically determine the significant differences between males and females of *D. citri* in low–temperature or normal–temperature (n.s. indicates no

significance) treatments. FNT and MNT: female and male under normal temperature (26°C); FLT and MLT: female and male under low temperature (15°C). Every group had four biological replicates of *D. citri*.
